# Influence of Post Material and Cement Type on the Retention of Prefabricated Posts in Endodontically Treated Teeth

**DOI:** 10.3390/ma19194041

**Published:** 2026-09-22

**Authors:** Damla Güneş Ünlü, Pınar Yıldız, Zekiye Eroğlu, Esra Talay Çevlik

**Affiliations:** 1 Department of Prosthodontics, Faculty of Dentistry, Hacı Bektaş Veli University, 50300 Nevşehir, Türkiye; drdamlaunlu@gmail.com (D.G.Ü.); zeroglu@nevsehir.edu.tr (Z.E.); 2 Nimet Bayraktar Oral and Dental Health Center, 38050 Kayseri, Türkiye; dentistpinar@hotmail.com; 3 Department of Prosthodontics, Faculty of Dentistry, Aydın Adnan Menderes University, 09100 Aydın, Türkiye

**Keywords:** PEEK, PEKK, glass-fiber post, pull-out test

## Abstract

Purpose: This *in vitro* study aimed to evaluate the effects of post material and cement type on the pull-out force of prefabricated glass-fiber, PEEK, and PEKK posts. Methods: Sixty extracted human mandibular premolars were prepared with standardized root canals and allocated into six groups (*n* = 10) according to post material (glass-fiber, PEEK, PEKK) and luting cement (resin or glass ionomer). PEEK and PEKK posts were CAD-CAM fabricated from prefabricated discs. All specimens underwent thermocycling (6000 cycles, 5–55 °C) prior to pull-out force testing on a universal testing machine, and failure modes were classified as adhesive, cohesive, or mixed. Because variance homogeneity was not satisfied, pull-out force values were logarithmically transformed (Log10) before two-way ANOVA and Bonferroni-adjusted post hoc analyses. Results: Post material (*p* = 0.026), cement type (*p* < 0.001), and their interaction (*p* = 0.012) significantly influenced pull-out force. Under resin cement, no statistically significant differences were detected among glass-fiber, PEEK, and PEKK posts. Under glass ionomer cement, PEEK and PEKK posts demonstrated significantly higher pull-out force than glass-fiber posts. The highest mean pull-out force was observed for glass-fiber posts luted with resin cement. The effect of post material on pull-out force was dependent on the luting cement. PEEK posts luted with resin cement showed no significant difference in pull-out force compared with conventional glass-fiber posts, supporting their potential as an alternative post material. Further studies are needed to evaluate the clinical performance of PEEK posts.

## 1. Introduction

Fiber-reinforced resin and glass-fiber posts have been widely regarded as favorable alternatives to metal posts in the rehabilitation of endodontically treated teeth because of their superior esthetics, more homogeneous stress distribution, biocompatibility, corrosion resistance, ease of manipulation, and ability to bond to resin-based restorative materials [1,2,3,4]. Nevertheless, despite these advantages, fiber posts may concentrate mechanical stresses in the cervical dentin and restoration margins and do not inherently reinforce the remaining tooth structure [5]. Moreover, although their elastic modulus (45.7–53.8 GPa) is lower than that of metal posts (95.0–110.0 GPa), it remains substantially higher than that of dentin (18.6 GPa), which may contribute to biomechanical incompatibilities [6]. Another important limitation is their relatively weak adhesion to root canal dentin and the potential for post dislodgement, which can subsequently result in microleakage and restoration failure [5,7].

Polyether ether ketone (PEEK) and polyether ketone ketone (PEKK) are high-performance thermoplastic polymers belonging to the polyaryl ether ketone (PAEK) family and are known for their favorable physicochemical properties. One of the principal advantages of PEEK and PEKK is their compressive strength and elastic modulus (3–4 GPa [8] and 5.1 GPa [9], respectively), which are close to those of dentin, enabling effective stress distribution and shock absorption within the restored tooth structure. In addition, these materials exhibit several desirable characteristics, including low plaque affinity, good biocompatibility, lightweight structure, and favorable biomechanical behavior [10,11,12]. Their relatively low elastic modulus and viscoelastic behavior may contribute to a more favorable stress distribution and reduced stress concentration at the post–dentin interface compared with more rigid post materials. Furthermore, their favorable mechanical properties and suitability for CAD-CAM fabrication make PEEK and PEKK attractive candidates for the development of standardized or customized intraradicular post systems. Notably, PEKK has been reported to provide further performance benefits within the PAEK family, exhibiting superior physical and mechanical characteristics—particularly higher compressive strength—compared with PEEK [13,14]. However, the clinical performance of intraradicular post systems depends not only on their mechanical properties but also on adequate bonding to root canal dentin. Despite the growing interest in PAEK materials, evidence regarding their bonding performance remains limited, particularly for PEKK posts. Therefore, the aim of this study was to evaluate the effects of post material and luting cement on pull-out force.

The null hypotheses were that post material, luting cement, and their interaction would have no effect on pull-out force.

## 2. Materials and Methods

This study was approved by the Non-Interventional Clinical Research Ethics Committee of Erciyes University (Approval Number: 2019/858). Extracted teeth obtained during routine dental treatments were anonymised and included with the informed consent of the patients.

To address the factorial design of the study, an a priori power analysis was conducted using G*Power software (Version 3.1.9.7) specifically for a two-way ANOVA (Fixed effects, special, main effects and interactions). To detect a large effect size (Cohen’s f = 0.42) for both the main effects and the interaction effect, with an alpha error probability of 0.05, six experimental groups, and a numerator degrees of freedom of 2, the calculation indicated that a minimum total sample size of 58 was required to achieve a statistical power of 80.0%. To ensure equal distribution across all subgroups, the final total sample size was rounded up to 60 (*n* = 10 per group) [10,15,16].

Sixty human mandibular premolar teeth were selected based on radiographic and clinical examinations, with inclusion criteria requiring a single, negotiable root canal with a mature apex and straight root morphology, and absence of root caries, previous endodontic treatment, internal calcifications, or resorption. After removal of calculus and soft tissue, the teeth were stored in 0.1% thymol solution (Thymol; Supelco^®^, Sigma-Aldrich Chemie GmbH, St. Louis, MO, USA) at room temperature for three months. Crowns were sectioned perpendicular to the long axis near the cementoenamel junction to standardize root length at 14 mm.

A single operator (D.U.) performed all root canal preparations manually using a step-back technique with a standardized working length of 14 mm. Canals were irrigated with 2 mL of 5.25% NaOCl after each instrument, then rinsed with distilled water, dried, and obturated using gutta-percha with the lateral condensation technique and root canal sealer (AH-Plus; Dentsply DeTrey GmbH, Konstanz, Germany). Specimens were stored in distilled water for one week. Post-space preparation was performed conventionally, leaving 4 mm of gutta-percha for the apical seal and achieving a post space length of 10 mm, using drills provided by the fiber post system manufacturer (3M ESPE, St. Paul, MN, USA, lot no. 036010609). Canals were irrigated with distilled water, dried with paper points (Ocean Dental, Istanbul, Turkey), and each post was trial-fitted prior to cementation.

In the present study, three types of materials—glass-fiber (3M ESPE, USA), PEEK (PEEK-OPTIMA Dental Disc, Juvora, Lancastershire, UK, lot no. J000025), and PEKK (Pekkton^®^, Cendres + Metaux, Milan, Italia, lot no. 200368) —were evaluated as dental post systems. PEEK and PEKK posts were fabricated using a CAD-CAM system (Amann Girrbach AG, Koblach, Austria) by scanning a prefabricated size 2 glass-fiber post (1.6 mm apical diameter, 0.80 mm coronal diameter, 20 mm length, and 4.5° taper), converting it into a digital model with a laboratory scanner, and milling 20 posts per material from filled discs using the milling unit (Figure 1).

Sixty human premolar teeth were randomly allocated into six experimental groups (*n* = 10 per group) based on the post material and the type of luting cement used (Table 1). Glass-fiber posts were assigned to two groups according to the luting cement used: FR, cemented with a dual-cure resin cement (RelyX U200; 3M ESPE, St. Paul, MN, USA, lot no. 4819681), and FC, cemented with a glass ionomer cement (Meron; VOCO, Cuxhaven, Germany, lot no. 581122). Similarly, PEEK posts were allocated to PR (resin cement) and PC (glass ionomer cement) groups, while PEKK posts were allocated to PKR (resin cement) and PKC (glass ionomer cement) groups.

All root canals were cleaned with 96% ethanol, irrigated with saline, and dried prior to cementation. Luting of all posts was performed with dual-cure resin cement and glass ionomer cement according to the manufacturer’s instructions. The glass-fiber posts were used directly from the manufacturer’s package without any additional surface treatment, including cleaning, silanization, primer application, or other pretreatments. No surface treatment was applied to the PEEK or PEKK posts prior to cementation.

Prior to testing, specimens were immersed in distilled water and subjected to 6000 thermocycles between 5 and 55 °C with a 30 s dwell time and 6 s transfer time. Pull-out force tests were conducted on a universal testing machine (Model 8872; Instron Corp., Norwood, MA, USA) at a crosshead speed of 0.5 mm/min until failure. Specimens were mounted in the jig with the loading direction parallel to the post lateral surface. The maximum force causing post-debonding was recorded in Newtons (N) (Figure 2).

Following mechanical testing, failure modes were assessed under a stereomicroscope (×20 magnification) by a single calibrated examiner (D.U.). Failures were classified according to the location of failure as adhesive (Type 1), cohesive (Type 2), or mixed (Type 3).

IBM SPSS Statistics for Windows (Version 26.0; IBM Corp., Armonk, NY, USA) was used to conduct statistical analyses. Levene’s test was used to assess homogeneity of variance, and the Shapiro–Wilk test was used to examine the distribution of the data. Because substantial variance heterogeneity was observed, particularly in the glass-fiber/resin cement group, pull-out force values were logarithmically transformed (Log10) prior to inferential analysis. A two-way analysis of variance (ANOVA) was then used to evaluate the effects of post material, cement type, and their interaction on pull-out force. Because the interaction between post material and cement type was significant, simple main effects and pairwise comparisons were examined using Bonferroni-adjusted tests. A significance level of *p* < 0.05 was used to determine statistical significance. Descriptive statistics are reported using the original, untransformed pull-out force values.

## 3. Results

Descriptive statistics and two-way ANOVA test results for the pull-out force values of all experimental groups are presented in Table 2.

The main effect of post material was statistically significant (F = 3.891, *p* = 0.026, ηp^2^ = 0.126), as was the main effect of cement type (F = 19.148, *p* < 0.001, ηp^2^ = 0.262). A significant interaction was observed between post material and cement type (F = 4.809, *p* = 0.012, ηp^2^ = 0.151), indicating that the effect of cement type varied according to post material.

Because the interaction between post material and cement type was significant, simple main effects were examined. When resin cement was used, no statistically significant differences were detected among glass-fiber, PEEK, and PEKK posts (F = 1.894, *p* = 0.170). In contrast, significant differences among post materials were observed when glass ionomer cement was used (F = 18.912, *p* < 0.001). PEEK posts exhibited significantly higher pull-out force than both PEKK (*p* = 0.006) and glass-fiber posts (*p* < 0.001), while PEKK posts also demonstrated significantly higher pull-out force than glass-fiber posts (*p* = 0.032). Comparisons between cement types within each post material showed that resin cement resulted in significantly higher pull-out force than glass ionomer cement for glass-fiber posts (*p* = 0.001). No statistically significant difference between cement types was detected for PEEK (*p* = 0.133) or PEKK (*p* = 0.238).

Among all experimental groups, the highest mean pull-out force value was recorded in the glass-fiber–resin cement group (139.59 ± 101.31 N), whereas the lowest value was observed in the glass-fiber–glass ionomer cement group (33.85 ± 7.77 N).

Type 1 failure was defined as an adhesive failure characterized by debonding of the post from the root canal walls; this failure mode was observed in the FC and PC groups. Type 2 failure was defined as cohesive failure within the post material, characterized by deformation (post deformation during loading). This was the most frequently observed failure mode and was detected in all specimens of the PR group. Type 3 failure was defined as a mixed failure pattern, in which specimens exhibited both deformation and/or fracture of the post material in conjunction with dislodgement from the root canal. This failure mode was predominantly observed in the FC, PKC, and PKR groups (Table 3) (Figure 3). Figure 4 shows the deformation of the post following testing compared with its original, undeformed configuration.

## 4. Discussion

The present study compared the pull-out force of PEEK and PEKK posts with that of conventional glass-fiber posts using two different luting cements. The main effects of post material and cement type were statistically significant, and a significant interaction between these factors was observed, indicating that the effect of post material depended on the luting cement. Under resin cement, no statistically significant differences were detected among glass-fiber, PEEK, and PEKK posts, although glass-fiber posts showed the highest numerical mean pull-out force. In contrast, under glass ionomer cement, PEEK posts demonstrated significantly higher pull-out force than both PEKK and glass-fiber posts, while PEKK also demonstrated significantly higher pull-out force than glass-fiber posts. These findings indicate that the relative retention performance of the post materials cannot be interpreted independently of the luting cement. Importantly, no statistically significant difference in pull-out force was detected between PEEK and conventional glass-fiber posts under the resin-cement condition. This finding suggests that PEEK may warrant further investigation as an alternative to conventional glass-fiber posts; however, equivalence cannot be concluded from the present findings.

Consistent with previous literature, a systematic review and meta-analysis study has reported that the mechanical and physical properties of PEEK posts are comparable to those of glass-fiber post–cores [17]. While numerous studies have primarily focused on the fracture strength of post materials [9,18,19,20,21], only a limited number have assessed bond strength using pull-out or push-out testing [10,18,22], and even fewer incorporated thermocycling into the experimental design [9,18,22]. In line with this methodological approach, the present study employed the pull-out test to evaluate the retention performance of the posts, which provides a comprehensive assessment of overall retention performance in prefabricated and CAD-CAM post-and-core restorations. Unlike the push-out method, which may be influenced by artifacts related to specimen sectioning, pull-out testing better reflects the clinical dislodgement behavior of posts.

Saisho et al. [18] reported that PEEK post–core specimens predominantly showed deformation rather than fracture compared with glass-fiber, polymer ceramic, and composite resin groups. Consistent with these findings, the PEEK specimens in the present study also exhibited deformation without catastrophic failure. Notably, all resin-cemented PEEK post specimens showed cohesive failure accompanied by post deformation during loading, which may be associated with the intrinsic viscoelastic characteristics of PEEK rather than being solely attributable to the adhesive interface.

An *in vitro* study by Benli et al. [10] and a finite element analysis by Lee et al. [23] identified PEEK and PEKK, respectively, as promising alternatives to conventional post systems. In addition, Ghanem et al. [14] reported the clinical performance of intraradicular PEKK posts comparable to that of fiber posts in a pilot randomized controlled trial. Consistent with these previous findings [10,14,23], PEEK posts luted with resin cement exhibited pull-out force that was not statistically different from that of glass-fiber posts in the present study. However, the present findings provide additional evidence that the performance of these materials is cement-dependent. Under GIC, both PEEK and PEKK demonstrated significantly higher pull-out force than glass-fiber posts, whereas no significant differences among the three materials were detected under resin cement. The contrasting material rankings between the two cement conditions may partly explain the significant interaction observed in the present study. Given the limited evidence currently available regarding PEKK posts, these findings should be interpreted with caution. Moreover, direct evidence regarding the retention behavior of PEEK and PEKK posts under different luting cement conditions remains limited, making direct comparison with previous studies challenging. Therefore, the present findings should be considered as preliminary evidence that may help guide further investigations of PAEK-based intraradicular posts.

El-Gawad et al. [24] reported that conventional cementation protocols remain clinically applicable. In line with this perspective, both resin and glass ionomer cements were included to compare different luting strategies for glass-fiber and PAEK posts. To the best of the authors’ knowledge, no previous study has directly compared the pull-out force of PAEK posts cemented with resin and glass ionomer cements. In the present study, cement type showed a significant overall effect on pull-out force; however, the significant post material × cement interaction indicates that this effect should be interpreted according to post material. Resin cement produced significantly higher pull-out force than GIC for glass-fiber posts, whereas no statistically significant difference between the two cements was detected for PEEK or PEKK. Importantly, the numerical pull-out force of PEEK was lower with GIC than with resin cement (73.74 versus 108.65 N), despite the absence of a statistically significant difference (*p* = 0.133). Therefore, the lack of statistical significance should not be interpreted as equivalence between the two cements. Rather, the findings indicate that no statistically significant difference between the two cements was detected for PEEK under the conditions tested. Failure mode analysis supported the material- and cement-dependent nature of the debonding behavior. Adhesive failures at the post–dentin interface (Type 1) were observed only in the glass ionomer cement groups (FC and PC), whereas cohesive failure within the post (Type 2) was the predominant failure mode in the PR group. Mixed failures (Type 3) occurred most frequently in the PKR and PKC groups, suggesting that the debonding behavior of PAEK posts may be influenced by both the luting cement and the mechanical response of the post material under tensile loading. The observed failure patterns may also reflect differences in the adhesive interaction between the post surfaces and the luting cements, as well as possible changes in cement properties following thermocycling. However, because the present study did not separately evaluate post–cement bond strength or the mechanical properties of the cements before and after thermocycling, these factors cannot be established as direct causes of the observed failure modes.

The differences observed among the post–cement combinations may be related to the interaction between the mechanical characteristics of the post materials and the bonding behavior of the luting cements. The relatively flexible and viscoelastic nature of PEEK and PEKK may allow greater deformation during tensile loading, potentially affecting the way stresses are transferred through the post–cement–dentin complex. In addition, differences in surface characteristics and chemical compatibility between the PAEK materials and the luting cements may have contributed to the different retention patterns observed between the resin cement and glass ionomer cement groups. However, these factors were not independently evaluated in the present study and therefore represent possible explanations rather than demonstrated mechanisms.

This study has several limitations. First, PEEK and PEKK posts were fabricated as prefabricated posts with dimensions comparable to those of conventional glass-fiber posts to ensure standardization. However, these materials can also be manufactured using customized CAD/CAM workflows or different prefabrication techniques, which may alter their surface characteristics and geometry. Therefore, the present findings are limited to the post design evaluated in this study. In addition, no surface treatment was applied to the PAEK posts prior to cementation. Since surface modification has been reported to enhance the pull-out force of PAEK materials, future studies should investigate the effects of different surface treatment protocols on the retention of these post systems. Furthermore, surface roughness, actual surface area, canal-post adaptation, and cement film thickness were not quantitatively evaluated, which may have influenced the pull-out force.

## 5. Conclusions

- PEEK posts luted with resin cement showed no significant difference in pull-out force compared with conventional glass-fiber posts.
- With GIC, PEEK and PEKK showed significantly higher pull-out forces than glass-fiber posts.
- The significant post material × cement interaction indicates that the retention performance of these materials depends on the luting cement used.
- These findings support further investigation of PEEK as an alternative to conventional glass-fiber posts, particularly when resin cement is used.

## Figures and Tables

**Figure 1:**
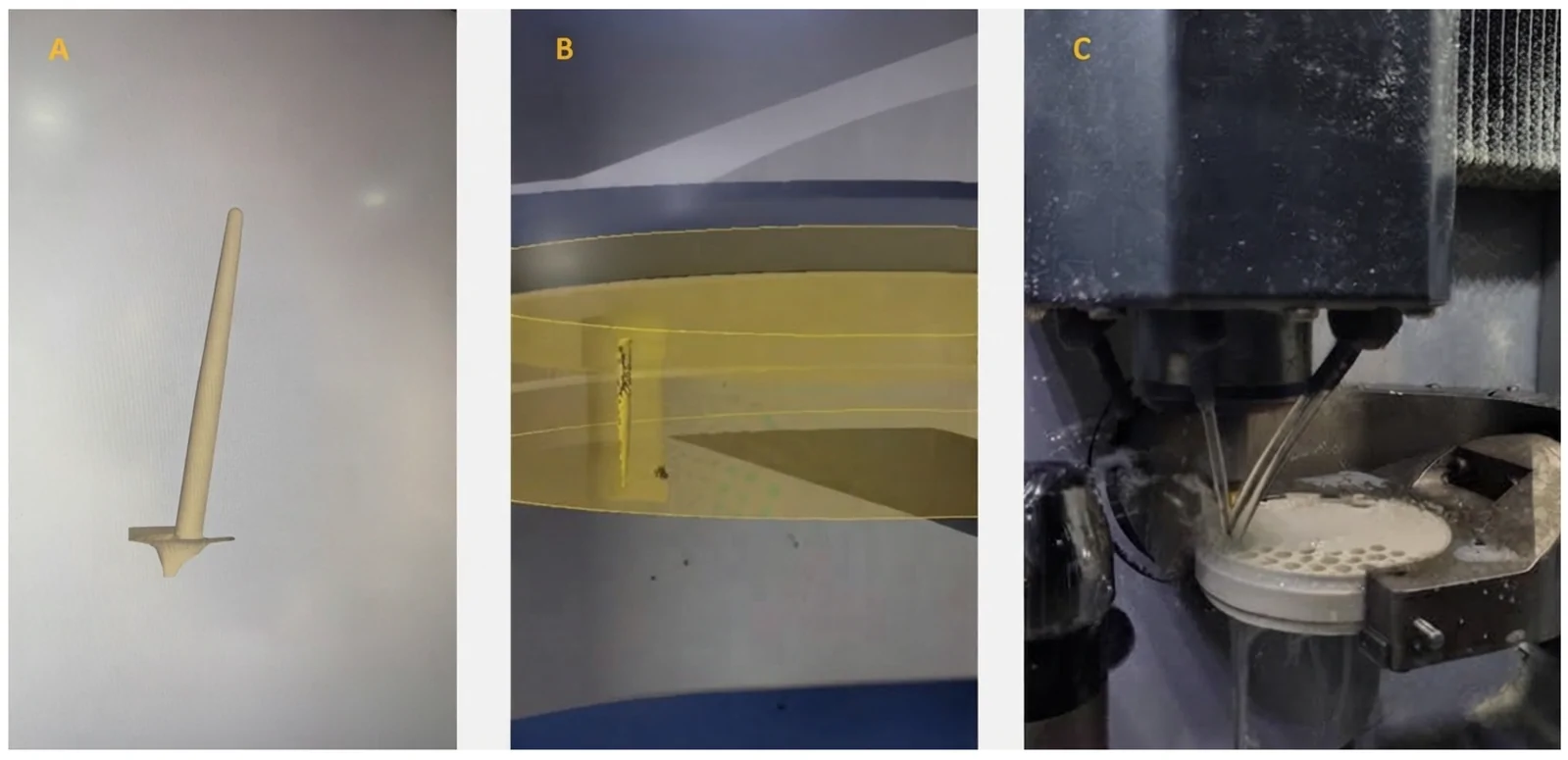
Design and production of PEEK and PEKK posts. (**A**) Digital design of the scanned reference post; (**B**) positioning of the post design within the material disc; (**C**) CAD-CAM milling of the posts.

**Figure 2:**
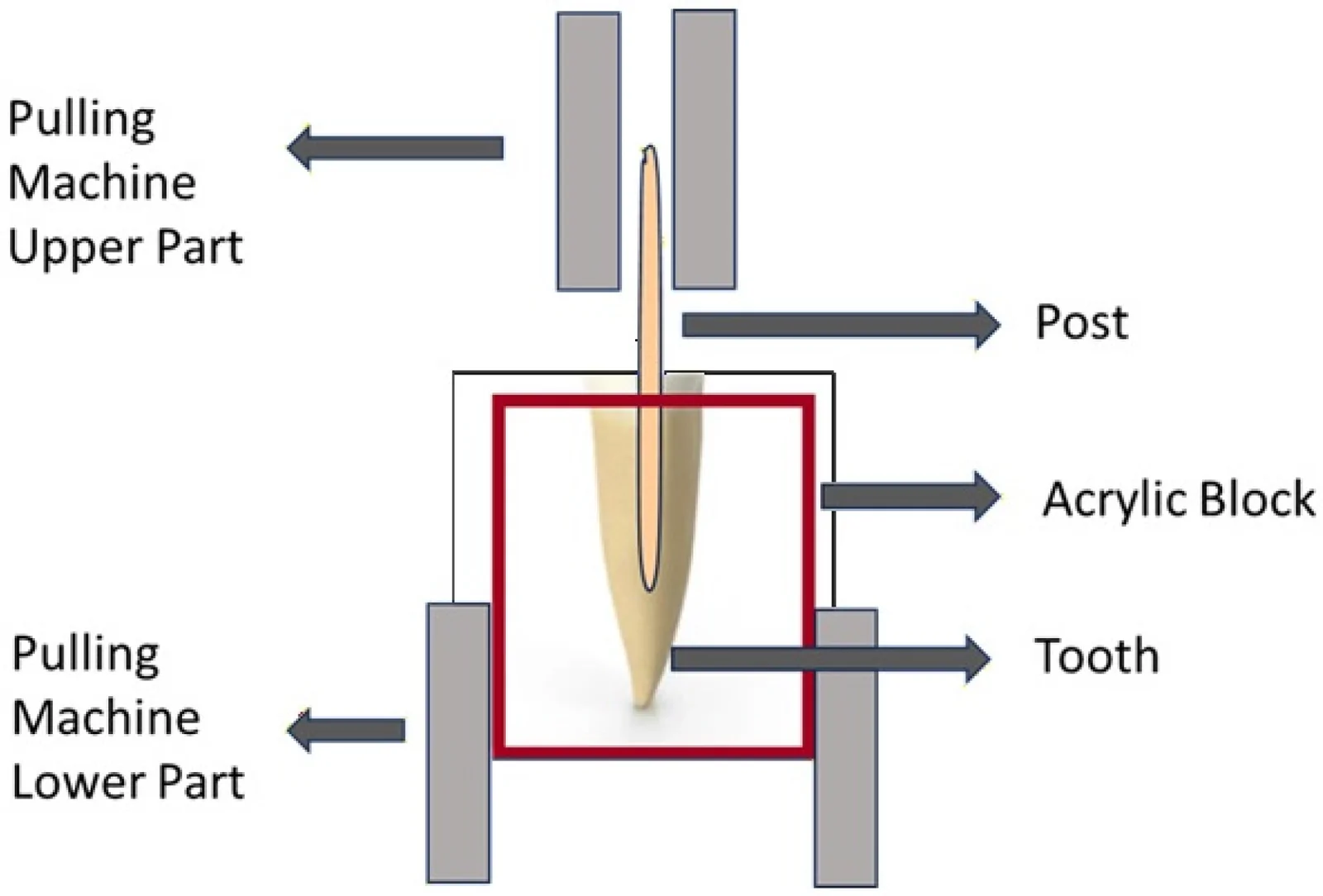
Diagrammed representation of the pull-out force test.

**Figure 3:**
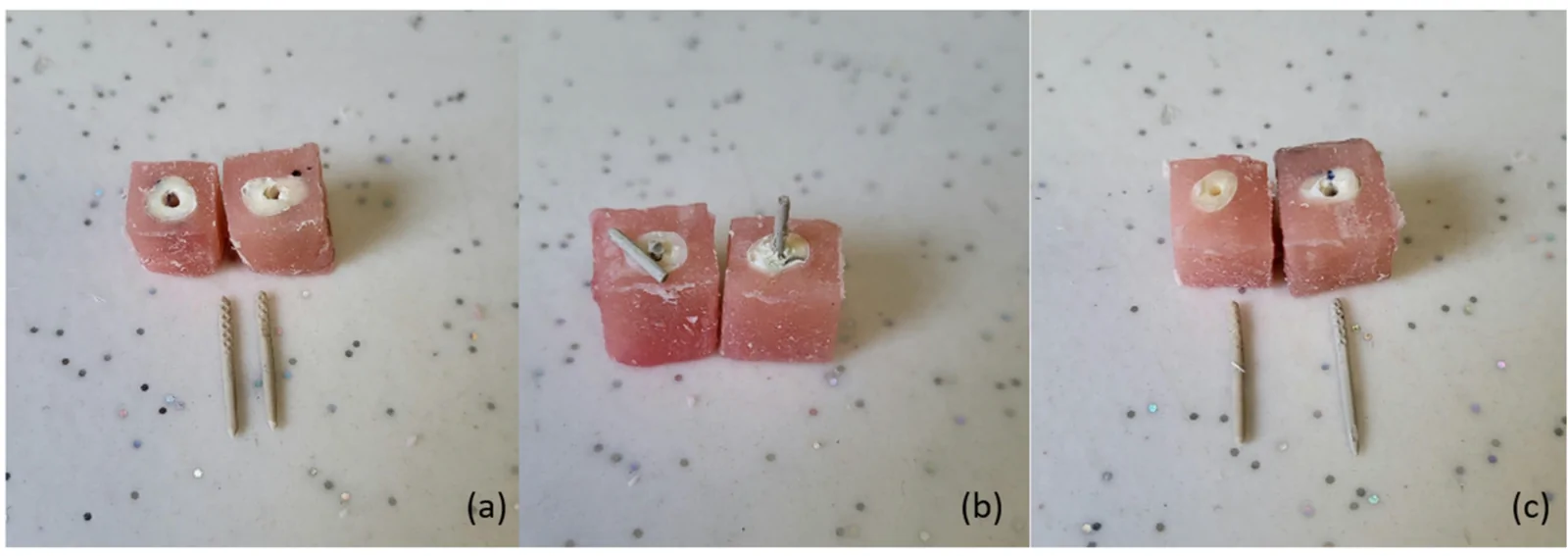
Type of failures (**a**) Type 1 (adhesive failure): debonding of the post from the root canal walls; (**b**) Type 2 (cohesive failure): post deformation during loading; (**c**) Type 3 (mixed failure): specimens exhibited both deformation and/or fracture of the post material in conjunction with dislodgement from the root canal.

**Figure 4:**
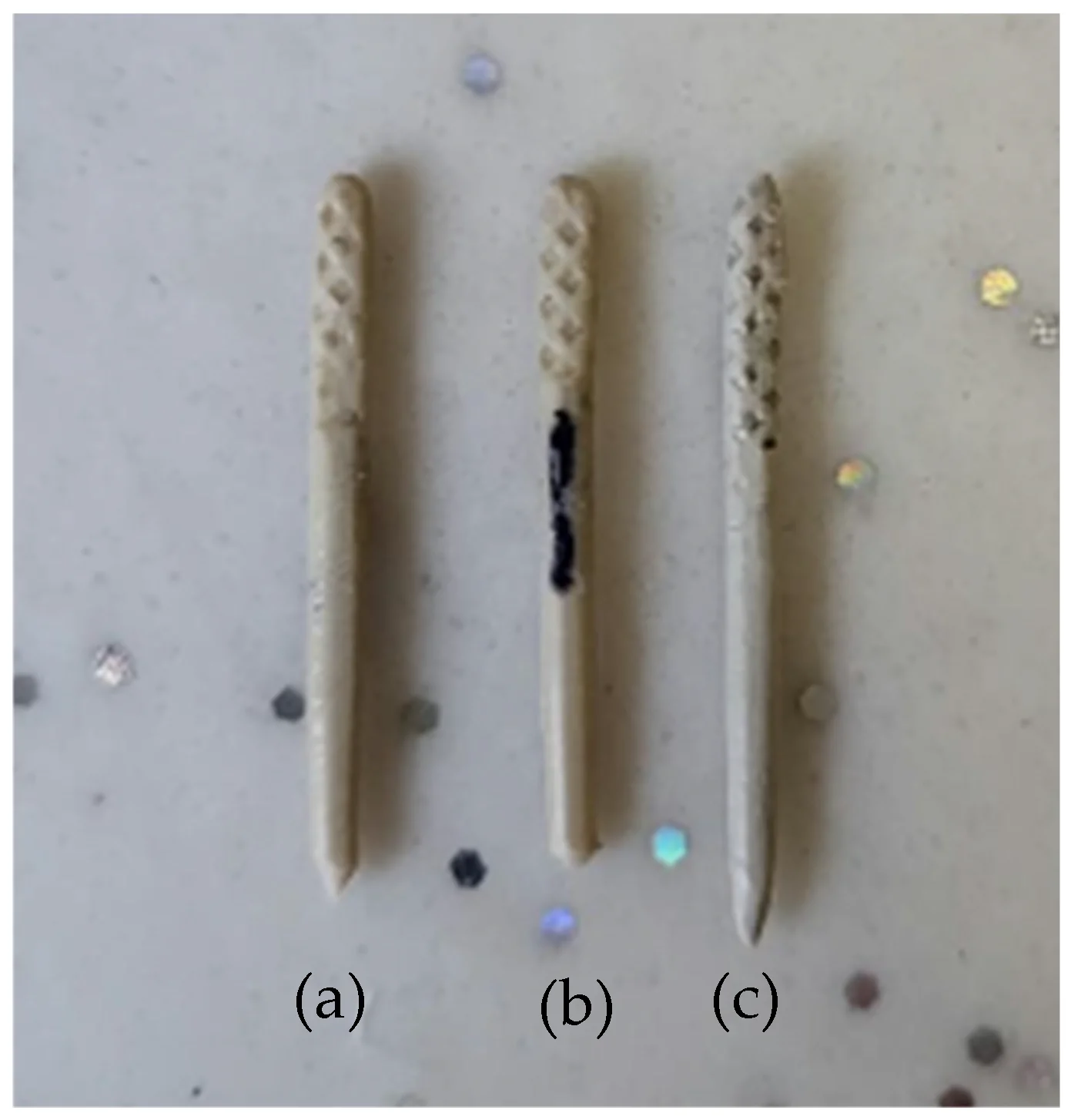
Representative post conditions following testing: (**a**) adhesive failure without visible post deformation, (**b**) post fracture, and (**c**) post deformation.

**Table 1:** The specimens, fabrication methods and milling details.

Group	Post Material	Fabrication Method	Milling Details	Cementation
FR	Glass-fiber	Prefabricated	–	Self-adhesive resin cement
FC	Glass-fiber	Prefabricated	-	Glass Ionomer cement
PR	PEEK	CAD-CAM	Scanned from group F, 20 posts milled from PEEK-OPTIMA disc	Self-adhesive resin cement
PC	PEEK	CAD-CAM	Same as PR	Glass Ionomer cement
PKR	PEKK	CAD-CAM	Scanned from group F, 20 posts milled from PEKK—Pekkton^®^ disc	Self-adhesive resin cement
PKC	PEKK	CAD-CAM	Same as PKR	Glass Ionomer cement

**Table 2:** Two-way ANOVA and Bonferroni test results of the pull-out test.

	Material			Test Statistics ^†^	
	Fiber	PEEK	PEKK		
	$\bar{x}$ **±** sd	$\bar{x}$ **±** sd	$\bar{x}$ **±** sd	**F**	***p***
**Cement**					
RESIN	139.59 ± 101.3086 ^A,x^	108.647 ± 53.4626 ^A,x^	70.939 ± 33.2922 ^A,x^	1.894	0.170
GIC	33.8450 ± 7.77027 ^B,x^	73.735 ± 19.9622 ^A,y^	47.963 ± 12.6045 ^A,z^	18.912	<0.001
**Test Statistics ^‡^**					
*F*	4.358	1.591	1.221		
*p*	0.001	0.133	0.238		

Mean, SD: standard deviations. ^†^ Group comparison between materials within the same cement type; different lowercase superscripts (x, y, z) in the same row indicate significant differences. ^‡^ Comparison between cements within the same material; different uppercase superscripts (A, B) in the same column indicate significant differences. Groups with the same superscripts are not significantly different (*p* > 0.05). Statistical analyses were performed on Log10-transformed pull-out force values; descriptive statistics are presented as original values.

**Table 3:** Percentages of failure modes in each experimental group.

Failure Mode	Groups					
	FR (n = 10)	FC (n = 10)	PR (n = 10)	PC (n = 10)	PKR (n = 10)	PKC (n = 10)
Type 1	0	5 (50%)	0	2 (20%)	0	0
Type 2	8 (80%)	0	10 (100%)	7 (70%)	2 (20%)	3 (30%)
Type 3	2 (20%)	5 (50%)	0	1 (10%)	8 (80%)	7 (70%)

## Data Availability

The original contributions presented in this study are included in the article. Further inquiries can be directed to the corresponding author.
